# Implementation of a cloud-based electronic patient-reported outcome (ePRO) platform in patients with advanced cancer

**DOI:** 10.1186/s41687-021-00358-2

**Published:** 2021-09-15

**Authors:** Olga Generalova, Mohana Roy, Evan Hall, Sumit A. Shah, Kristen Cunanan, Touran Fardeen, Brianna Velazquez, Gilbert Chu, Bianca Bruzzone, Anna Cabot, George A. Fisher, Sandy Srinivas, Alice C. Fan, Sigurdis Haraldsdottir, Heather A. Wakelee, Joel W. Neal, Sukhmani K. Padda, Tyler Johnson, Gregory M. Heestand, Robert W. Hsieh, Kavitha Ramchandran

**Affiliations:** 1 Stanford Cancer Institute, Stanford, USA; 2 Present Address: Division of Medical Oncology, Stanford University School of Medicine, Stanford, USA; 3 Department of Medical Oncology, University of Washington School of Medicine, Seattle, WA USA; 4 Clinical Research Division, Fred Hutchinson Cancer Research Center, Seattle, WA USA; 5 Quantitative Sciences Unit, Stanford University School of Medicine, Stanford, USA; 6 Stanford Healthcare, Stanford, USA

**Keywords:** Patient reported outcomes, Quality of life, Care-delivery, Healthcare utilization

## Abstract

**Background:**

Patient reported outcomes (PROs) have been associated with improved symptom management and quality of life in patients with cancer. However, the implementation of PROs in an academic clinical practice has not been thoroughly described. Here we report on the execution, feasibility and healthcare utilization outcomes of an electronic PRO (ePRO) application for cancer patients at an academic medical center.

**Methods:**

We conducted a randomized trial comparing an experimental ePRO arm to standard of care in patients with advanced cancer in the thoracic, gastrointestinal, and genitourinary oncology groups at Stanford Cancer Center from March 2018 to November 2019. We describe the pre-implementation, implementation, and post-implementation phases of the ePRO arm, technological barriers, electronic health record (EHR) integration, clinician burden, and patient data privacy and security. Feasibility was pre-specified to be at least 70% completion of all questionnaires. Acceptability was based on patient and clinician feedback. Ambulatory healthcare utilization was assessed by reviewing numbers of phone messages, electronic portal messages, and referrals for supportive care.

**Results:**

Of 617 ePRO questionnaires sent to 72 patients, 445 (72%) were completed. Most clinicians (87.5%) and patients (93%) felt neutral or positive about the ePRO tool’s ease of use. Exposure to ePRO did not cause a measurable change in ambulatory healthcare utilization, with a median of less than two phone messages and supportive care referrals, and 5–6 portal messages.

**Conclusions:**

Web-based ePRO tools for patients with advanced cancer are feasible and acceptable without increasing clinical burden. Key lessons include the importance of pilot testing, engagement of stakeholders at all levels, and the need for customization by disease group. Future directions for this work include completion of EHR integration, expansion to other centers, and development of integrated workflows for routine clinical practice.

**Supplementary Information:**

The online version contains supplementary material available at 10.1186/s41687-021-00358-2.

## Introduction

Prior studies of advanced cancer patients suggest that patient-reported symptom monitoring is associated with prolonged survival [1], improved communication with physicians, nurses [2, 3] and other members of the healthcare team [4], and decreased utilization of unplanned healthcare (e.g., emergency department visits) [5]. Therefore, measuring the patient experience through instruments such as Patient Reported Outcomes (PROs) has gained attention as a means of monitoring patients’ symptoms while also promoting patient engagement in their own care [6–9].

The advent of mobile technology has enabled several real-time, patient-initiated, symptom-tracking applications (electronic PROs or ePROs). However, there are a paucity of data for the implementation of ePROs in clinical practice [10]. While ePROs have been shown to improve quality of life, concerns arise about increased patient and clinician burden, low uptake, and limitations from electronic health records (EHRs). We conducted a randomized clinical trial for the introduction of ePRO at an academic medical center to: (1) identify key features that could be applied in general clinical practice, and (2) assess impact on healthcare utilization.

## Methods

We designed a clinical trial with an accrual goal of 144 subjects from the thoracic, genitourinary, and gastrointestinal disease groups at Stanford Cancer Center. Patients were randomized 1:1 to the experimental (ePRO) arm or the standard of care arm (“Appendix A”). Patients were age 18 years or older and English speaking with advanced cancer, an ECOG performance status of 0–2, and a life expectancy of at least 6 months (see “Appendix B” for full eligibility criteria). This report describes a subset of the clinical trial outcomes, including ePRO implementation, feasibility, and acceptability, and its effect on health care utilization. (a separate report will describe health-related quality of life as measured by the PROMIS-G [11] and FACT-G [12] instruments.) We defined patient feasibility a priori as greater than 70% completion of questionnaires, and defined patient acceptability as a neutral or higher satisfaction. We also measured symptom responses and ambulatory healthcare (HC) utilization. The latter was assessed by retrospective chart review of numbers of phone messages, electronic portal messages, and referrals to supportive care services (including social work, psycho-oncology, psychiatry) during the six-month trial period. Only patients who completed the full duration of the intervention as well as the week 24 questionnaire were assessed for HC utilization (43 in ePRO arm and 47 in the standard of care arm).

## Pre-implementation

### ePRO selection

The Noona platform is a cloud-based mobile service, designed to capture PROs in oncology. Other platforms were rejected because they were not compatible with EHR integration, oncology-specific issues, or utilization in the context of a clinical trial. This platform was selected because its validation as an ePRO tool, adaptability to institutional preferences, and inclusion of oncology-specific modules.

### EHR integration and data security

While the platform met requirements for future integration into our EHR platform, full integration was not implemented for the study period. The Data Risk Assessment Stanford Information Security Office evaluated the ePRO platform and recommended the following steps which were completed: (1) ensuring the platform complied with university minimum security standards, (2) configuring a secure two-step authentication system for all data users, (3) transferring all data with use of secure transmission protocols. All of these recommendations were listed with a moderate or high-risk level.

### Stakeholder and leadership engagement

Buy-in prior to the ePRO launch was prioritized. In 2015–2016, we obtained input and approval from the three oncology disease groups, the Information Technology department, the chief technical officer, and the director of operations. Focus groups included key personnel from the oncology disease groups, information technology and operations. These collaborations ensured buy-in of the ePRO tool with the goal of possible future clinical integration.

### Clinical workflow development

**Fig. 1:**
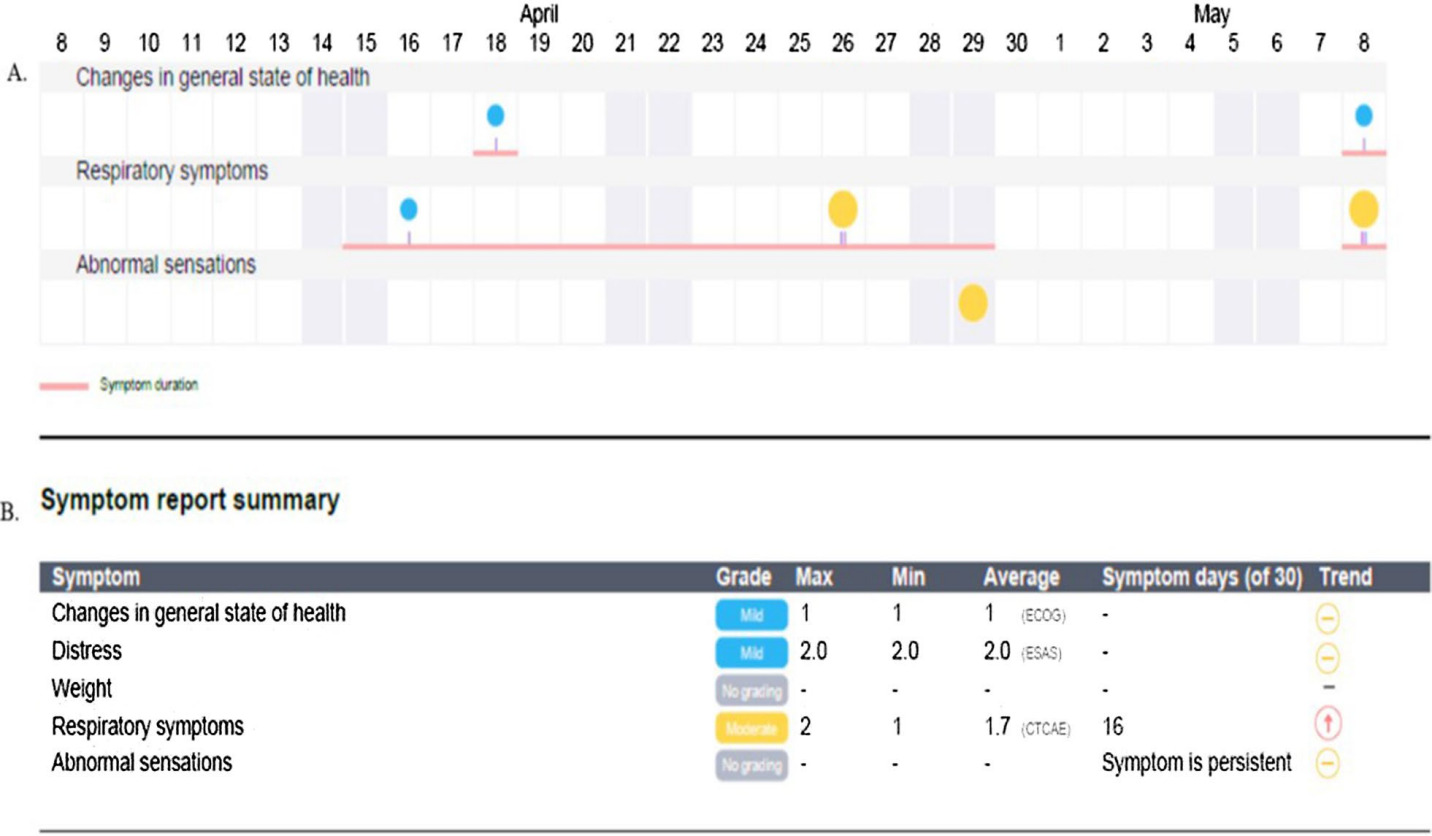
Visualization of symptoms—imeline and summary reports on ePRO platform. Panel **A** Calendar format for symptom type, severity, and duration. Panel **B** Report summary listing symptoms by grade, maximum and minimum values, duration, and improved, worsening, or stable trends

The clinical care teams of each oncology disease group included physicians, advanced practice providers (nurse practitioners and physician assistants), and registered nurses. These teams performed initial testing at Stanford Cancer Center (October 2016). They suggested thresholds for clinical intervention, frequency of assessments and a system to provide ePRO results to providers before the clinic visit. At routine intervals, patients received symptom questionnaires (SQs), in which they could select relevant symptom icons for symptom severity and duration (Fig. 1 and Additional file 1: Figure S1). We conducted three working sessions with clinical staff to establish symptom severity criteria and a consensus workflow for severe symptoms. The ePRO staff responded with iterative changes to the platform, which included the Edmonton Symptom Assessment System (ESAS) for assessment of depression and anxiety symptoms. The initial testing period allowed us to develop thresholds within the SQ for clinical intervention, customize the ePRO based on disease group, treatment schedule, and type of treatment (Additional file 1: Table S1).

Given that the platform, while EHR compatible, required initial validation per university standards, we developed a clinical workflow that would allow engagement with the tool and represent as integrated of a system as possible. The workflow involved patients filling out the SQ on the ePRO platform, and the CRC reviewing those symptoms and uploading the document to the EHR as a clinical note. A PDF file of the SQ also showed a calendar view and printout of the patient’s symptoms for providers for easy reference. The CRC also provided a verbal summary to the clinician prior to the visit. The EHR portal was still used for messaging for patients given its routine use in our clinical practice. The intended workflow ultimately would be more seamless with an EHR ePRO, that would not require such additional staff support. Twenty patients piloted the ePRO tool from November 2016 to January 2017, completing the required SQs and diary entries.

## Implementation

### Training for the research team

The research team consisted of the Primary Investigator and Clinical Research Coordinators (CRCs). The ePRO staff trained the research team with demonstration accounts to ensure familiarity with the ePRO prior to implementation. Bi-weekly study meetings of the research team with platform staff promoted a collaborative approach and facilitated troubleshooting.

### Clinical care team training and engagement

CRCs trained their respective clinical care teams to explain the eligibility criteria and intervention. The thoracic, gastrointestinal, and genitourinary clinical groups were enrolled consecutively, rather than simultaneously, allowing for individualized problem-solving to accommodate each clinic’s unique workflows.

### Patient onboarding

CRCs reviewed the study with each participant during the initial visit, and used the “Noona Patient User Manual” to demonstrate how to complete the SQ. The CRC educated patients on how to communicate symptoms with their clinical teams via e-secure messaging, phone, and/or the ePRO. Participants received instructions to contact the clinic directly or call 911 when experiencing a medical emergency and not use the ePRO tool to report symptoms requiring immediate medical attention.

### Symptom questionnaire

**Fig. 2:**
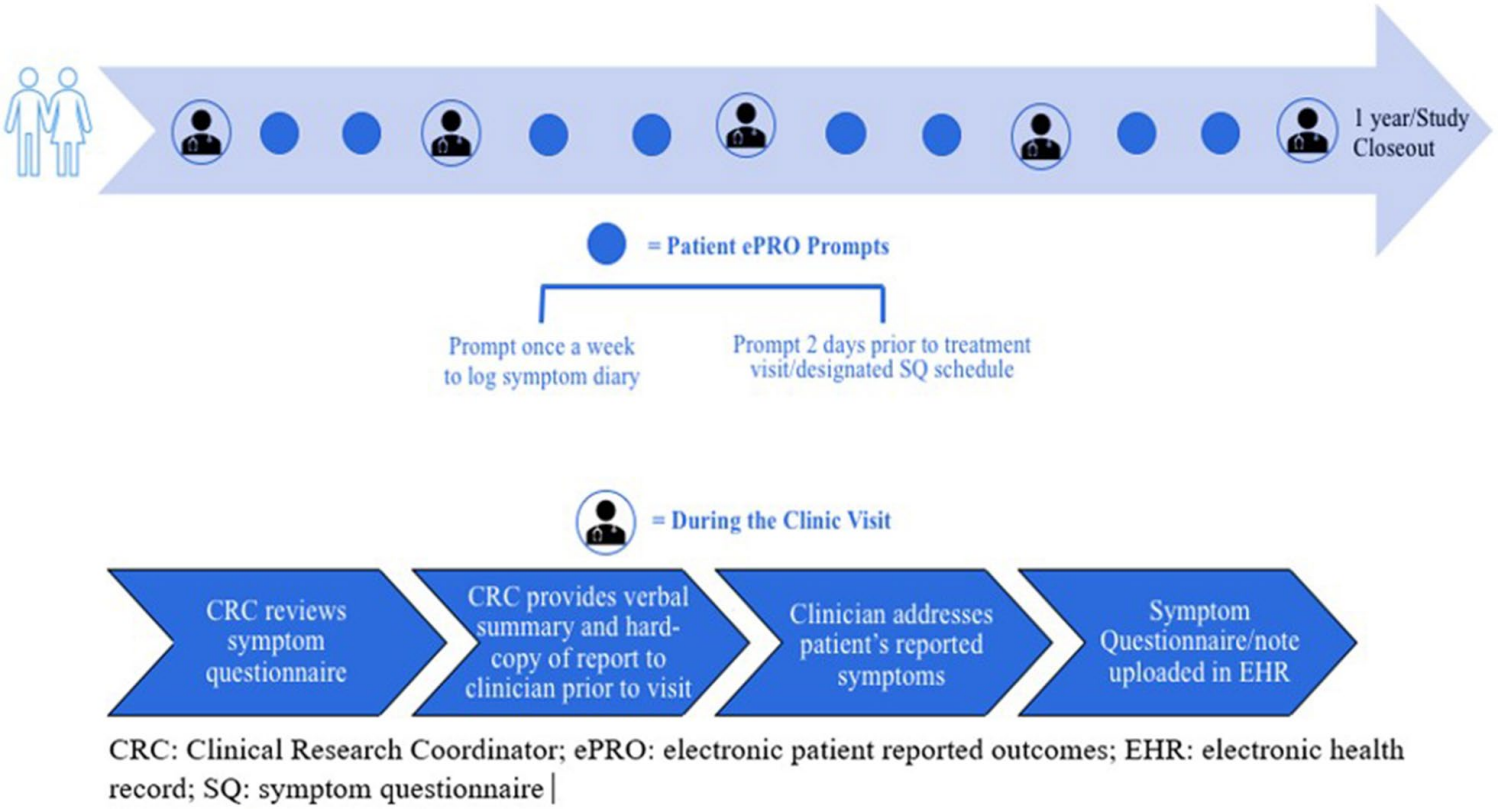
Schema of ePRO intervention. Overall schema of the ePRO intervention with symptom logs, questionnaires, and in-clinic review. For example, patients on cytotoxic regimens such as FOLFOX for colorectal cancer received infusions every two weeks and were thus prompted to complete a SQ every two weeks. Patients on oral medications might not be seen in clinic as frequently and were thus prompted to complete a SQ by a scheduled clinic visit and at a minimum of every three weeks

Patients received prompts to complete a SQ at least every three weeks or up to two days prior to upcoming oncology clinic visits (Fig. 2). If the patient failed to complete the SQ, the CRC sent a reminder via the EHR portal. Any symptom that met or exceeded criteria for a clinically severe symptom immediately generated an internal alert in the patient’s profile, which was relayed to the clinical team within one business day. Criteria for severe symptoms were defined using validated scales specific to each symptom type. Physical symptoms (not including pain) were graded based on the Common Terminology Criteria for Adverse Events (CTCAE v. 4.0). Pain was graded using a visual analog scale (VAS) from 0 to 10. Distress was graded using the Edmonton Symptom Assessment System (ESAS). Performance status was graded using the Eastern Cooperative Oncology Group (ECOG) scale. Severe symptoms were classified as CTCAE of grade 3 or 4 or pain score greater than VAS 7. Mild to moderate symptoms were communicated to the clinical team at the point of care via both printed and electronic summary reports. Clinical staff reviewed this report prior to the clinic encounter.

## Post implementation

### Feasibility and acceptability

The platform sent 617 digital SQs to the 72 patients randomized to the ePRO intervention arm. Of these invitations, 445 (72%) digital SQs were completed. This rate increased to 82% (419/512) after excluding patients who withdrew, passed away, or entered hospice care within the first 3 months of study enrollment. Qualitative feedback on patient and clinician satisfaction were collected at baseline, at the halfway mark, and at completion (“Appendix C” and “Appendix D”). Neutral or positive satisfaction was reported by 87.5% of clinicians (n = 28/32) and 93% of patients (n = 43/46) when asked if the tool was easy to use. Furthermore, 47% of clinicians (n = 15/32) and 56% of patients (n = 26/46) would probably or definitely recommend the platform to others. Examples of clinician observation regarding use of the platform included the “color coded” “graphic display”, which “allowed…patients to communicate with us in a different way”. Some patient observations included patients liking “having a diary” and communicating “symptoms…ahead of [their] appointment”. Constructive comments from clinicians involved issues surrounding lack of integration to the EHR and that the care team was already “fielding a lot of electronic communication” with most symptoms reported “at clinic or through standard messaging”. Patients noted software issues, with issues on “re-selecting each symptom” when entering data, with difficulty in addressing “all issues”, and without an option to select “no change” in symptoms.

### Patient-reported symptoms

The 72 patients in the ePRO arm reported a mean of 45 symptoms, a median of 40 symptoms, and a range of 1 to 72 symptoms during their 6-month period on the ePRO platform. The most reported symptoms were “overall change in health” (n = 518), “distress” (n = 358), “weight change” (n = 343), “fatigue” and “weakness” (n = 330), “gastrointestinal symptoms” (n = 308), and “pain” (n = 240). Patients reported a low frequency of severe symptoms: two patients had three severe symptoms in the VAS scale; 17 patients had 34 severe symptoms in the CTCAE (v. 4) scale; and 18 patients had 38 severe symptoms in the ESAS scale.

### Ambulatory healthcare utilization

Ninety patients from both arms of the trial completed a 24-week follow up questionnaire. These patients generated a median of 1–2 phone encounters, 5–6 electronic portal messages, and 0–1 supportive care referrals. These data showed no significant differences between the ePRO and control groups. The interquartile ranges were narrow, ranging from 0–1 for all three outcomes.

## Discussion

PRO tools are increasingly recognized as vital to understanding and managing patient symptoms in cancer care. While early studies have shown improvement in outcomes, there is minimal data on ePRO implementation. Our key findings were as follows.

A. Operations and implementation
  1. Support from organizational leadership and clinical teams at all levels, and the ePRO vendor ensured appropriate clinical workflows, clinician acceptability and follow-up.
  2. Initiation of ePRO benefitted from the scaffolding of a clinical trial protocol.
  3. Staged implementation of the ePRO platform allowed specific action items to be addressed and modified as needed.
B. Feasibility and acceptability
  1. Implementation of ePRO in an at-risk patient population was feasible and acceptable. The recommendation for the platform from both clinicians and patients was less than 50%, although satisfaction rates for ease of use were higher. We hypothesize that this discordance could be due to increased workflow for staff and clinicians, even with CRC support, given lack of EHR integration. As for patient satisfaction, possible reasons for lower recommendation rates include time burden of logging symptoms, issues with the platform (ex: need to log chronic symptoms repeatedly), and lack of integration directly with the primary patient facing portal. The higher satisfaction regarding usability likely reflects on the ePRO platform itself, as noted with example comments from clinicians and patients.
  2. Exposure to ePRO did not generate an increase in healthcare utilization.

The study team anticipated several implementation barriers. These included information technology, clinical workflow variations, missing data, patient attrition, patient privacy, academic and technology partnership, and regulatory processes. Adjusting for these barriers during the pre-implementation phase allowed for a more immediate response to troubleshooting.

One concern in the implementation of ePROs is the potential risk of increasing the burden on healthcare providers from the additional data generated by patients. For the purposes of the trial, this was mitigated by our research coordinator who highlighted ePRO findings for the clinicians and facilitated a timely means of documentation.

A second concern is that patients might report severe symptoms via the SQ in lieu of seeking immediate medical attention. To avoid this risk, we designed an algorithm for the platform that prompted patients to contact their medical team immediately or call 911 for severe symptoms. The CRCs also reported severe symptoms to the clinical team within one business day. Reassuringly, this workflow for acute symptom reporting did not increase healthcare provider burden, as measured by ambulatory healthcare utilization. Of note, the overall low numbers of phone and electronic portal messages, and severe symptoms reported, in both groups was surprising, but may reflect (a) prompt in-clinic management of symptoms and (b) patient hesitancy in reporting to a third-party platform. The latter point is an important limitation as ePRO platforms rely on patient trust in reporting personal information in a way that is not traditional in the patient-clinician relationship. Regarding monitoring cadence, we chose to include patients who were on active treatment and had frequent follow up, thus likely reporting symptoms at point of care. A future consideration is to expand to patients receiving less frequent care or perhaps those using telemedicine.

Another concern was over-representation of chronic symptoms. For instance, a patient experiencing chronic, severe fatigue while on treatment would need to manually re-enter this symptom on each successive SQ. This generated repeated alerts for the clinical teams. While inconvenient, this feature was preserved to maintain the study methodology. A future implementation might allow patients to define symptoms as “chronic” and flag only those symptoms that are new or concerning. Lastly, programmatic cost becomes a concern in such implementation, with an estimate of $100/patient, depending on the scope of services.

Our study of ePRO implementation in cancer care expands on previous studies by detailing several barriers to implementation that have been observed in prior reports. These include concerns about data privacy and security, increased health care provider burden, and overdocumentation of symptoms from patients [13–16]. This report describes an ePRO system from pre-implementation to successful study completion that proved to be acceptable and feasible in an academic cancer center. To our knowledge, this is one of few reported studies to describe ePRO implementation with a structured timeline of processes that can be replicated at other sites. Future directions include integration with our EHR, expansion to other locations in our health care system, inclusion of non-English speaking patients, further integration of workflows into routine clinical practice and enhanced patient empowerment and engagement.

### Supplementary Information

**Additional file 1.Table S1**: shows the sample schedule frequencies for symptom questionnaires sent to patients based on treatment type. **Figure S1**: shows a sample user interface of the ePRO platform, where patients can view there overall symptom scores (top left), can record type of symptom overall (top right), and fill out the specific symptoms in the symptom questionnaire (bottom panel).

## Data Availability

Not applicable to current manuscript, however datasets used during the current study are available from the corresponding author on reasonable request.

## Appendix B: Participant eligibility criteria

A Participant Eligibility Checklist must be completed in its entirety for each subject prior to registration. The completed, signed, and dated checklist must be retained in the patient’s study file and the study’s Regulatory Binder.

The study coordinator and treating physician must verify that the participant’s eligibility is accurate, complete, and legible in source records. A description of the eligibility verification process will be included in the EPIC or other Electronic Medical Record progress note.

### Inclusion criteria

**Subjects eligible for enrollment in the study must meet all of the following criteria:**

1. Individuals aged 18 years or older.
2. Access to smartphone, tablet or computer with capability to utilize symptom tracking application.
3. Advanced (recurrent or metastatic) lung or gastrointestinal cancer, or advanced renal, bladder or castrate resistant prostate cancer.
4. No limit on prior lines of therapy in the metastatic setting; must be on active treatment while enrolled in the study
5. ECOG performance status of 0–2
6. Estimated life expectancy of at least 6 months
7. Willing and able to comply with all study procedures.
8. Willing and able to provide written, signed informed consent after the nature of the study has been explained, and prior to any research-related procedures.

### Exclusion criteria

**Subjects meeting any of the following criteria must not be enrolled in the study:**

1. Concurrent disease or condition that interferes with participation or safety.
2. Non-English speaking, as the application is developed in the English language.
3. Non-castrate resistant prostate cancer.

## Appendix C: Clinician satisfaction survey

1. 1. Do you consider Noona to be a valuable addition to the care of your cancer patients?
  **Table:**
  Absolutely not		Neutral		Definitely
  1	2	3	4	5
2. Would you recommend Noona to a colleague?
  **Table:**
  Absolutely not		Neutral		Definitely
  1	2	3	4	5
3. Would you be interested in continuing to use Noona in your practice after the completion of the study?
  **Table:**
  Not at all		Neutral		Definitely
  1	2	3	4	5
4. Do you feel that the use of Noona presented a significant additional burden to you and your care team?
  **Table:**
  Not at all		Neutral		Definitely
  1	2	3	4	5
5. How often did you review Noona data for your patients?
  **Table:**
  Not at all	Rarely	Sometimes	Often	At every visit
  1	2	3	4	5
6. How often did you discuss Noona data with your patients?
  **Table:**
  Not at all	Rarely	Sometimes	Often	At every visit
  1	2	3	4	5
7. Did you feel that Noona data accurately reflected the experiences and symptoms of your patients?
  **Table:**
  Not at all		Neutral		Definitely
  1	2	3	4	5
8. Did you feel that Noona was easy to use?
  **Table:**
  Not at all		Neutral		Definitely
  1	2	3	4	5
9. I enjoyed the following other aspects of Noona:
10. I did not enjoy the following aspects of Noona:

## Appendix D: Patient feedback questionnaire

**Patient Feedback Questionnaire**

*Please answer the following questions based on your experience throughout the past three (3) months. You can choose to skip questions you do not wish to answer.*

1. Have you had any unplanned visits to the emergency department and/or an urgent care in the past 3 months?
  **Table:**
  Yes	□
  No	□
  If yes, how many?	
2. Please indicate your level of agreement with the following statement:
  In general, it is easy for me to communicate my symptoms with my care team.
  **Table:**
  Strongly agree	□
  Somewhat agree	□
  Neutral	□
  Somewhat disagree	□
  Strongly disagree	□
3. **Noona Use**
  1. In general, how often have you used Noona in the last month?
    **Table:**
    More than once a day	□
    Once a day	□
    Three to six times per week	□
    One to two times per week	□
    Rarely	□
    Never	□
  2. Which devices do you use to access Noona? (select all that apply)
    **Table:**
    Desktop computer	□
    Laptop computer	□
    Mobile phone	□
    Tablet	□
  3. Who was the primary user of the Noona tool?
    **Table:**
    Me. I am a patient at Stanford Cancer Institute	□
    A single caregiver	□
    Multiple caregivers	□
    Other	□
    If “Other”, please describe	
4. **Getting Started with Noona**
  **Please indicate your level of agreement with the following statement:**
  1. The Noona application was easy to use.
    **Table:**
    Strongly agree	□
    Somewhat agree	□
    Neutral	□
    Somewhat disagree	□
    Strongly disagree	□
  2. It was easy to log in to Noona.
    **Table:**
    Strongly agree	□
    Somewhat agree	□
    Neutral	□
    Somewhat disagree	□
    Strongly disagree	□
  3. The instructions provided by the study personnel on how to use Noona were helpful.
    **Table:**
    Strongly agree	□
    Somewhat agree	□
    Neutral	□
    Somewhat disagree	□
    Strongly disagree	□
  4. The instructions provided within the Noona tool were helpful when completing Symptom Questionnaires and the Symptom Diary.
    **Table:**
    Strongly agree	□
    Somewhat agree	□
    Neutral	□
    Somewhat disagree	□
    Strongly disagree	□
5. **Symptom Questionnaires**
  1. Did you receive electronic reminders from Noona to fill out questionnaires?
    **Table:**
    Yes	□
    No	□
    No, I did not elect to receive reminders	□
  2. Have you answered a Symptom Questionnaire (SQ) sent to you through Noona in the last 3 months?
    **Table:**
    Yes, I have answered all the questionnaires in my inbox	□
    Yes, but I have not answered all questionnaires in my inbox	□
    No, I have not answered any questionnaires in my inbox	□
    If “No,” why not?	
  3. It was easy to respond to a Symptom Questionnaire.
    **Table:**
    Strongly agree	□
    Somewhat agree	□
    Neutral	□
    Somewhat disagree	□
    Strongly disagree	□
  4. If you did not complete all of the Symptom Questionnaires, please describe why:
6. **Overall User Experience**
  1. Please rate the ease of using Noona to log your symptoms.
    **Table:**
    Very easy	□
    Somewhat easy	□
    Neutral	□
    Somewhat difficult	□
    Very difficult	□
  **Please indicate your level of agreement with the following statement:**
  2. Noona improved my ability to track my symptoms.
    **Table:**
    Strongly agree	□
    Somewhat agree	□
    Neutral	□
    Somewhat disagree	□
    Strongly disagree	□
  3. Noona helped my care team manage my symptoms related to my cancer.
    **Table:**
    Strongly agree	□
    Somewhat agree	□
    Neutral	□
    Somewhat disagree	□
    Strongly disagree	□
  4. Noona had a positive impact on my quality of life.
    **Table:**
    Strongly agree	□
    Somewhat agree	□
    Neutral	□
    Somewhat disagree	□
    Strongly disagree	□
  5. Noona helped me communicate with my care team.
    **Table:**
    Strongly agree	□
    Somewhat agree	□
    Neutral	□
    Somewhat disagree	□
    Strongly disagree	□
  6. Noona was time consuming to use.
    **Table:**
    Strongly agree	□
    Somewhat agree	□
    Neutral	□
    Somewhat disagree	□
    Strongly disagree	□
  7. My caregivers and family feel that Noona was useful for my care.
    **Table:**
    Strongly agree	□
    Somewhat agree	□
    Neutral	□
    Somewhat disagree	□
    Strongly disagree	□
    Not applicable	□
  8. Automatic reminders from Noona disruptive.
    **Table:**
    Strongly agree	□
    Somewhat agree	□
    Neutral	□
    Somewhat disagree	□
    Strongly disagree	□
  9. The use of Noona had a negative impact on my relationship with my cancer team.
    **Table:**
    Strongly agree	□
    Somewhat agree	□
    Neutral	□
    Somewhat disagree	□
    Strongly disagree	□
  10. Overall, I am satisfied with the Noona tool for symptom tracking.
    **Table:**
    Strongly agree	□
    Somewhat agree	□
    Neutral	□
    Somewhat disagree	□
    Strongly disagree	□
  11. If given the opportunity, I would continue to use Noona in my cancer care.
    **Table:**
    Strongly agree	□
    Somewhat agree	□
    Neutral	□
    Somewhat disagree	□
    Strongly disagree	□
  12. I would recommend Noona to other patients with a similar diagnosis.
    **Table:**
    Strongly agree	□
    Somewhat agree	□
    Neutral	□
    Somewhat disagree	□
    Strongly disagree	□
  13. If you feel that using Noona has influenced your illness, your treatment, or quality of life, please describe why.
  14. Do you have additional comments and/or suggestions?

## Abbreviations

CRC: Clinical Research Coordinator
EHR: Electronic health record
ePRO: Electronic patient reported outcome
SQ: Symptom questionnaire

## References

[1] Basch E, Deal AM, Dueck AC (2017). Overall survival results of a trial assessing patient-reported outcomes for symptom monitoring during routine cancer treatment. JAMA - J Am Med Assoc.

[2] Detmar SB, Muller MJ, Schornagel JH, Wever LDV, Aaronson NK (2002). Health-related quality-of-life assessments and patient-physician communication: a randomized controlled trial. J Am Med Assoc.

[3] Velikova G, Booth L, Smith AB (2004). Measuring quality of life in routine oncology practice improves communication and patient well-being: A randomized controlled trial. J Clin Oncol.

[4] Hilarius DL, Kloeg PH, Gundy CM, Aaronson NK (2008). Use of health-related quality-of-life assessments in daily clinical oncology nursing practice: a community hospital-based intervention study. Cancer.

[5] Basch E, Deal AM, Kris MG (2016). Symptom monitoring with patient-reported outcomes during routine cancer treatment: a randomized controlled trial. J Clin Oncol.

[6] Atkinson TM, Ryan SJ, Bennett AV (2016). The association between clinician-based common terminology criteria for adverse events (CTCAE) and patient-reported outcomes (PRO): a systematic review. Support Care Cancer.

[7] Fromme EK, Eilers KM, Mori M, Hsieh YC, Beer TM (2004). How accurate is clinician reporting of chemotherapy adverse effects? A comparison with patient-reported symptoms from the Quality-of-Life Questionnaire C30. J Clin Oncol.

[8] Di Maio M, Gallo C, Leighl NB (2015). Symptomatic toxicities experienced during anticancer treatment: agreement between patient and physician reporting in three randomized trials. J Clin Oncol.

[9] Basch E, Jia X, Heller G (2009). Adverse symptom event reporting by patients vs clinicians: relationships with clinical outcomes. J Natl Cancer Inst.

[10] Anatchkova M, Donelson SM, Skalicky AM, McHorney CA, Jagun D, Whiteley J (2018). Exploring the implementation of patient-reported outcome measures in cancer care: need for more real-world evidence results in the peer reviewed literature. J Patient-Reported Outcomes.

[11] Cella D, Riley W, Stone A (2010). The patient-reported outcomes measurement information system (PROMIS) developed and tested its first wave of adult self-reported health outcome item banks: 2005–2008. J Clin Epidemiol.

[12] King MT, Stockler MR, Cella DF (2010). Meta-analysis provides evidence-based effect sizes for a cancer-specific quality-of-life questionnaire, the FACT-G. J Clin Epidemiol.

[13] Hartkopf AD, Graf J, Simoes E (2017). Electronic-based patient-reported outcomes: willingness, needs, and barriers in adjuvant and metastatic breast cancer patients. JMIR Cancer.

[14] Karsten MM, Speiser D, Hartmann C (2018). Web-based patient-reported outcomes using the international consortium for health outcome measurement dataset in a major German university hospital: observational study. JMIR Cancer.

[15] Foster A, Croot L, Brazier J, Harris J, O’Cathain A (2018). The facilitators and barriers to implementing patient reported outcome measures in organisations delivering health related services: a systematic review of reviews. J Patient-Reported Outcomes.

[16] Biber J, Ose D, Reese J (2018). Patient reported outcomes—experiences with implementation in a University Health Care setting. J Patient-Reported Outcomes.

