# Supplementary material for: Implementation of a cloud-based electronic patient-reported outcome (ePRO) platform in patients with advanced cancer
Source: J Patient Rep Outcomes. 2021 Sep 15;5:91. doi: 10.1186/s41687-021-00358-2 (PMC8443731; doi:10.1186/s41687-021-00358-2)
Supplement: Supplementary file 1 — Additional file 1.Table S1: shows the sample schedule frequencies for symptom questionnaires sent to patients based on treatment type. Figure S1: shows a sample user interface of the ePRO platform, where patients can view there overall symptom scores (top left), can record type of symptom overall (top right), and fill out the specific symptoms in the symptom questionnaire (bottom panel). [file 41687_2021_358_MOESM1_ESM.docx]

**Data Supplements**

Table S1: Sample schedule frequencies for symptom questionnaires based on type of therapy.

| **Treatment Schedule Name** | **Cadence** |
| --- | --- |
| Systemic Therapy (e.g., Chemotherapy or Immunotherapy) 2 week cycle, 6 months | Every 2 weeks |
| Systemic Therapy (e.g., Chemotherapy or Immunotherapy) 3 week cycle, 6 months | Every 3 weeks |
| Systemic Therapy (e.g., Chemotherapy or Immunotherapy) 4 week cycle, 6 months | Every 2 weeks |
| Oral medicine treatment, 6 months | Baseline, on average every 3 weeks |


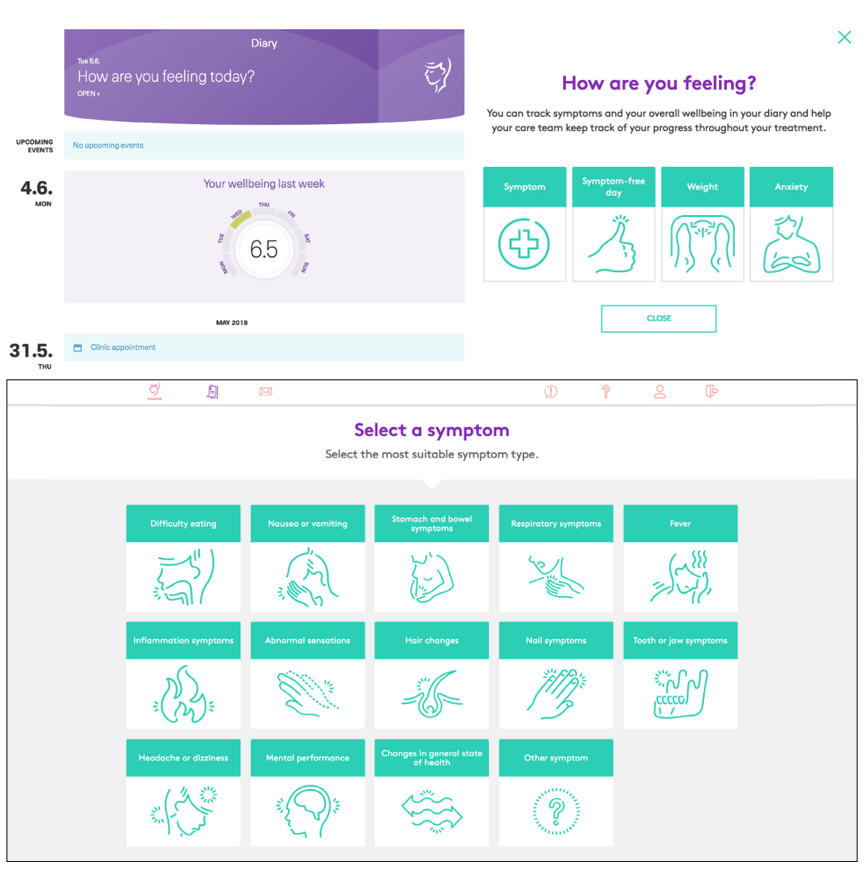
Figure S1: Sample Symptom Questionnaire (SQ) User Interface
